# Converting a Microarray Signature into a Diagnostic Test: A Trial of Custom 74 Gene Array for Clarification and Prediction the Prognosis of Gastric Cancer

**DOI:** 10.1371/journal.pone.0081561

**Published:** 2013-12-03

**Authors:** Ying Yin, Wei Zhuo, Yuan Zhao, Shujie Chen, Jun Li, Lan Wang, Tianhua Zhou, Jian-Min Si

**Affiliations:** 1 Department of Gastroenterology, Sir Runrun Shaw Hospital, School of Medicine, Zhejiang University, Hangzhou, China; 2 Institute of Gastroenterology, Zhejiang University, Hangzhou, China; 3 Department of Cell Biology and Program in Molecular Cell Biology, School of Medicine, Zhejiang University, Hangzhou, China; Oklahoma Medical Research Foundation, United States of America

## Abstract

**Background:**

Gastric cancer (GC) is associated with high mortality rates and an unfavorable prognosis at advanced stages. In addition, there are no effective methods for diagnosing gastric cancer at an early stage or for predicting the outcome for the purpose of selecting patient-specific treatment options. Therefore, it is important to investigate new methods for GC diagnosis.

**Methodology/Principal Findings:**

To facilitate its use in a diagnostic setting, a group of 74 genes with diagnostic and prognostic information was translated into a customized microarray containing a reduced set of 1,042 probes suitable for high throughput processing. In this report, we demonstrate for the first time that the custom mini-array can be used as a reliable diagnostic tool in gastric cancer. With an AUC value of 0.565 (95% CI 0.305-0.825) indicating a perfect test, the sensitivity and specificity of diagnosis from the ROC curve were calculated to be 70% and 80%, respectively.

**Conclusions/Significance:**

The data clearly demonstrate the reproducibility and robustness of the small custom-made microarray. The array is an excellent tool for classifying and predicting the outcome of disease in gastric cancer patients.

## Introduction

Gastric cancer (GC) has a high incidence and is the second leading cause of cancer mortality [1,2]. The prognosis of GC is highly dependent on the stage of the disease at diagnosis and the treatment method [3]. The 5-year survival rate in advanced gastric cancer patients is about 20%, whereas in early-stage gastric cancer it is above 60% [4-6]. However, there have been no effective and feasible methods for detecting early stage cancer and for predicting the possible prognosis to provide suitable treatment for each patient. In Japan, though they could more efficiently detect and treat early gastric cancer (EGC) through extensive screening, only endoscopy could be commonly used for the detection EGC. This is why early diagnosis and the ability to distinguish malignant and premalignant lesions are important [7]. Therefore, it is important to investigate new methods for GC diagnostic or prognostic predictions for clinical applications. 

Certain gene alterations could be associated with canceration and progression of GC [8-11]. For example, previous data we studied suggested that collagen genes might be a potential biomarker to distinguish malignant from premalignant lesions in the stomach [12]. Because the progression of disease from premalignant conditions to GC is a dynamic process [13-15], the detection of gene alterations could allow identification of disease-associated genes earlier than pathological examinations. In addition, gene expression provides additional information about a patient’s condition [16]. Therefore, microarray analysis may be an important and useful method for diagnosis and risk stratification in gastric cancer.

In addition, using customized mini-arrays for clinical practice may not only be cheaper but may also require less sample RNA input for labeling and hybridization, and the data processing time could be substantially reduced compared with normal microarrays [17]. A custom microarray of 70 genes for the prognosis of breast cancer, which has been approved by the Food and Drug Administration (FDA), verifies the feasibility of custom microarray in clinical use [18,19]. Although there have been several studies on certain groups of genes for GC diagnosis, the microarray technology is presently not used as a diagnostic tool in gastric cancer.

In this paper, we describe for the first time the development of a customized diagnostic GC mini-array and demonstrate that the custom mini-array could be used as a reliable diagnostic and prediction tool in gastric cancer. 

## Results

### A custom mini-array of a group of possible genes related to GC canceration and progress

In a previous study, we used an oligonucleotide microarray of 38,500 genes to systematically examine differential gene expression among 33 samples from normal, premalignant, and malignant lesions in the stomach [12]. A fraction of 696 differentiated-expression genes found in the formal study were designed in the custom mini-array as part of a research base. In addition, for some groups in which genes were found that were possibly closely related to GC, the custom mini-array also included 44 collagen related genes [20-22], 54 genes for sex hormone receptors and pathways, differentiated-expression genes found in other studies, and 915 normalization genes (detailed data in table S1). In this study, a 1042-gene expression profile was established as a powerful diagnosis and predictor of disease outcome in gastric cancer patients. 

### Comparison of the 1042-Gene Array Performance with That of Original 25k Microarray

**Figure 1:**
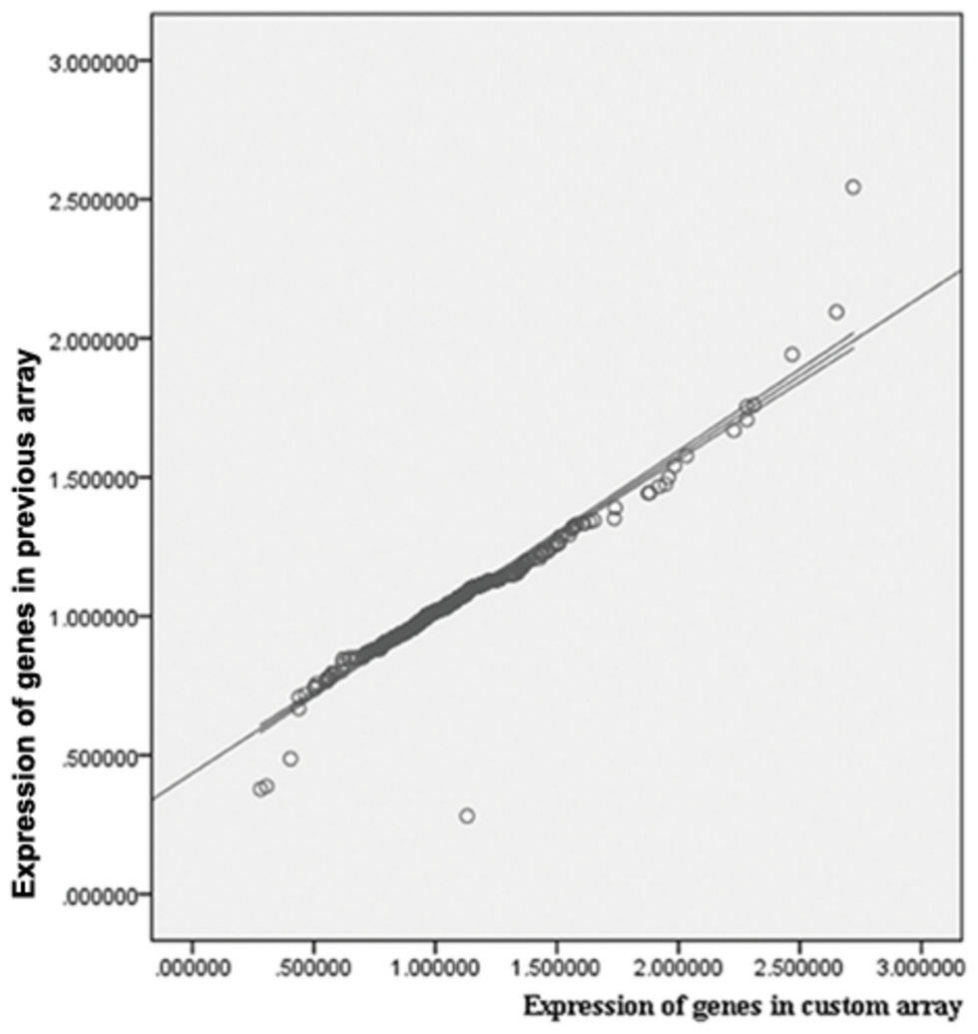
Expression data of custom microarray compared to previous study of the whole genes array with the same patient. The data indicated the expression ratio of malignant/premalignant. There were no significant differences between two groups.

To determine whether the customized mini-array test performs similar to the original 25 k microarrays [12], two samples (LYXT and LYXS) from a same patients used in the original series to develop the 1042-diagnostic classifier were retrieved. The expressions of 696 genes generated using the diagnostic mini-array were highly similar (Pearson correlation of 0.957, p < 0.01) to the original published data (Figure 1).

### Identification of genes differentially expressed in malignant and premalignant gastric tissues

**Figure 2:**
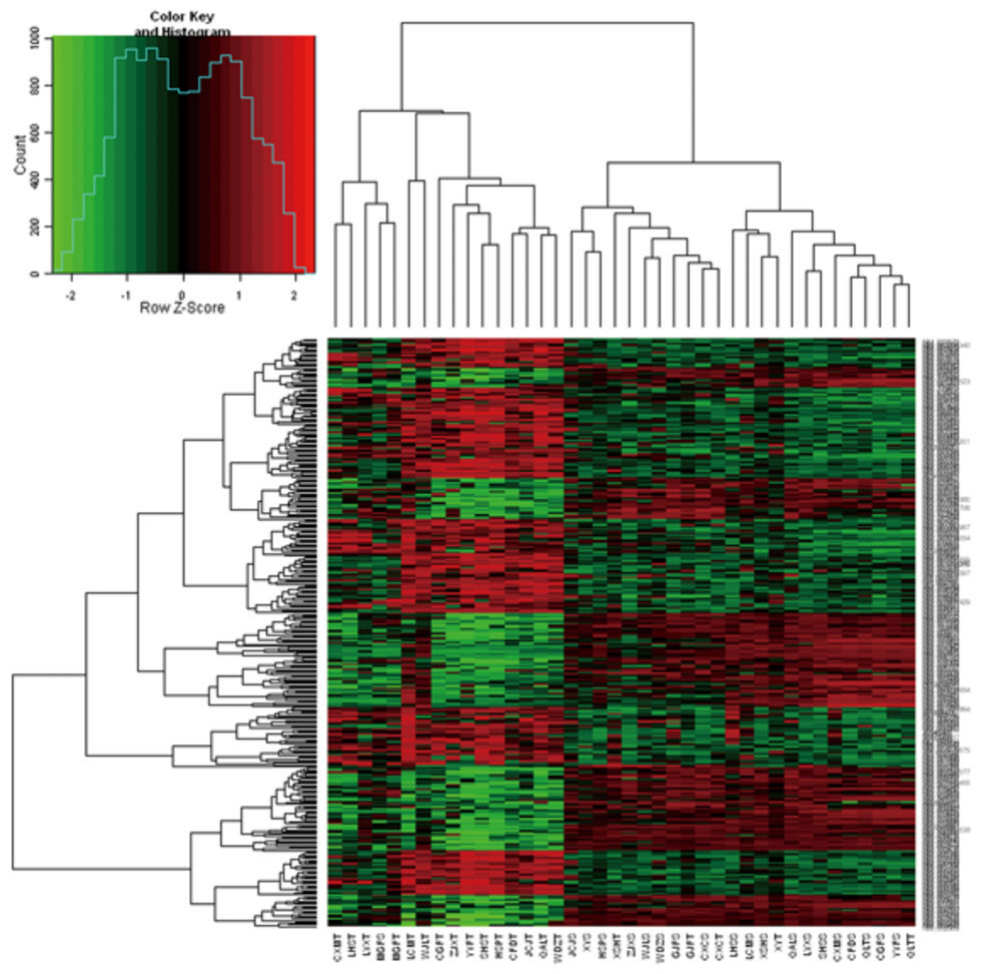
Expression data of genes from tumors and non-tumor specimens of 20 gastric cancer patients hybridized using the custom microarray of differential expression genes between two groups. Each column represents a sample and each row a gene. The character of “T” refers to tumor and the character of “S” refers to premalignant tissue in sample names. The expressions of genes between two groups have significant differences, fold change log_2_ >=1 or <= -1, P<0.01.

All data of hybridizations were background-corrected, normalized, and analyzed to identify the differentiated-expression genes in 40 samples that represent malignant lesions and premalignant lesions (n=20 in each group). A set of 371 genes was found to separate malignant lesions from premalignant lesions using hierarchical clustering and SVM leave-one-out confirmation, whereas a premalignant sample (MGFS) were classified into the malignant group, and five malignant samples (XSHT, GJFT, CXCT, XYT and QLTT) were classified into the premalignant group. The MGFS and sample was collected from the surrounding tissue of a signet-ring carcinoma. The pathological report revealed that the XSHT sample was a Moderately differentiated adenocarcinoma, the GJFT sample was a Moderately to well differentiated adenocarcinoma, whereas CXCT, XYT and QLTT samples were well differentiated adenocarcinoma. These differentially expressed genes included 199 up-regulated genes and 172 down-regulated genes in malignant lesions compared with premalignant lesions (Figure 2).

### Distinguishing the prognosis of gastric cancer

**Figure 3:**
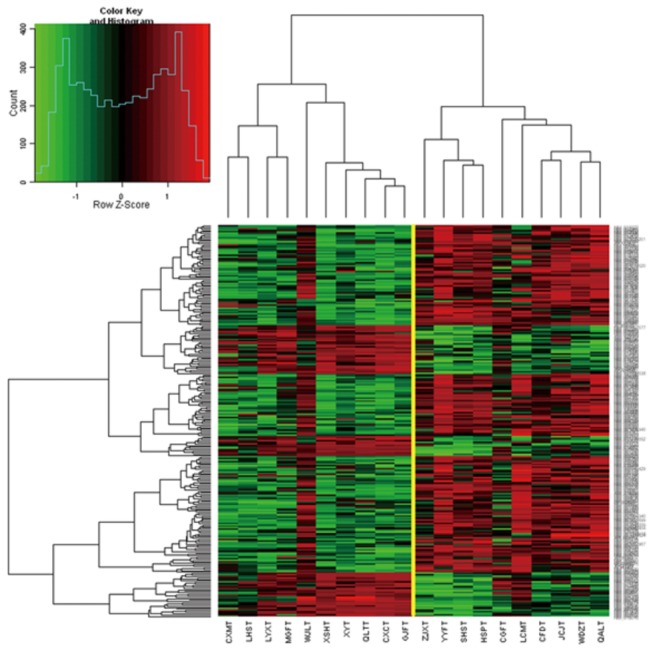
Expression data matrix of 371 potential prognostic markers genes from tumors of 20 gastric cancer patients hybridized using the custom microarray. Each column represents a tumor and each row a gene. The length and the subdivision of the branches display the relatedness of the GC (bottom) and the expression of the genes (right). The yellow line marks the subdivision into two dominant clusters. The expressions of genes between two groups have significant differences, fold change log_2_ >=1 or <= -1, P<0.01.

An unsupervised, hierarchical clustering algorithm allowed us to cluster the 20 GC malignant lesions on the basis of their similarities measured over significant genes of 371 differentially expressed genes between malignant and premalignant lesions. Notably, in the left group, 4 of 10 sporadic patients were at an early stage of GC and the others presented with highly differentiated lesions, whereas in the right group, 2 of 10 sporadic patients were from the group that developed distant metastases within 5 years or with high stage and poorly differentiated lesions. Thus, using unsupervised clustering, we can distinguish between 'good prognosis' and 'poor prognosis' tumors to some extent (Figure 3). In addition, the type and stage of GC of the patients were associated with the sub-groups of poor or good prognosis (Table 1, detailed data in table S2).

**Table 1:** The stage of gastric cancer patients in two groups.

	Group 1 (Good prognosis)	Group 2 (Poor prognosis)
Age	62.8±7.11	61.3±7.02
Gender (Male/Female)	7/3	8/2
Stage(I/II/III/IV)	2/1/7/0	1/0/5/4
Pathological type (Moderately to well differentiated adenocarcinoma / Poorly differentiated adenocarcinoma/ Signet ring cell carcinoma/ Mucinous adenocarcinoma)	7/2/1/0	3/4/1/2
Metastasis with 5 years	1	3

### Custom array with minimum number of genes for GC diagnosis and prediction

Unsupervised two-dimensional cluster analysis of gene clustering and GC clustering was performed independently using an agglomerative hierarchical clustering algorithm with 371 genes that could identify malignant GC and premalignant lesions. The correlation coefficient of the expression for each gene with disease outcome was calculated, and 252 genes were found to be significantly associated with disease outcome (correlation coefficient <-0.3 or >0.3) (detailed data in table S1). 

**Figure 4:**
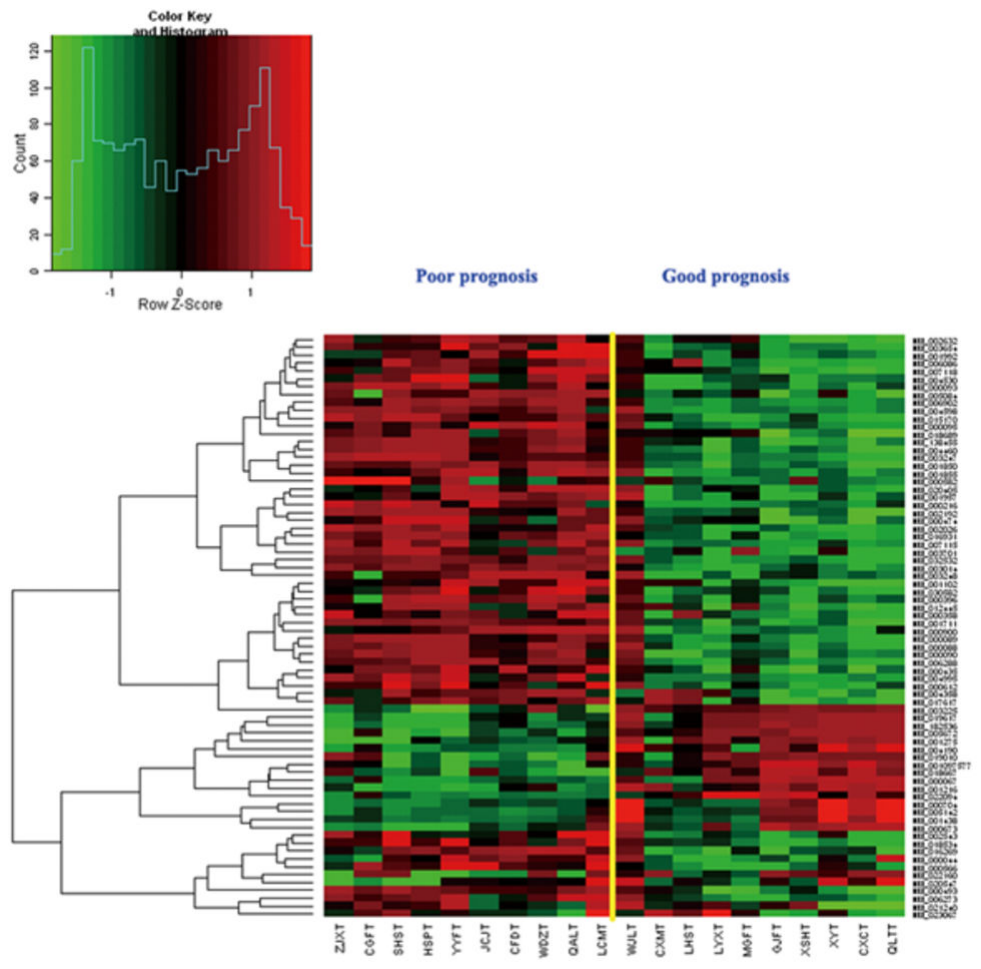
Expression data of final 74 custom microarray genes in 20 gastric cancer patients. Each column represents a tumor and each row a gene.

**Figure 5:**
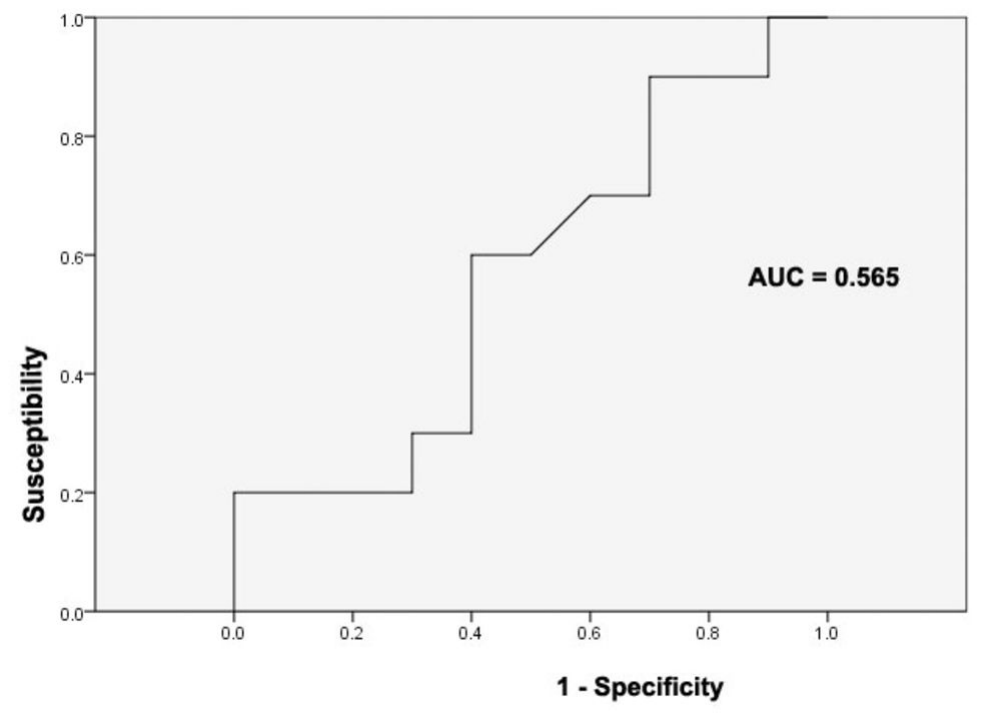
The ROC curve of the power of the classification for prognosis of 74 genes custom array.

These 252 genes were rank-ordered on the basis of the magnitude of the correlation coefficient. The number of genes in the 'prognostic classifier' was optimized by sequentially adding subsets of 5 genes from the top of this rank-ordered list and evaluating its power for correct classification using the 'leave-one-out' method for cross-validation. Classification was made based on the correlation of the expression levels of the remaining samples from the good and poor patients, respectively. The accuracy improved until the optimal number of marker genes was reached. Therefore, 74 genes were determined to be the minimum number of genes that could be classified as two sub groups of different prognosis (Figure 4). With an AUC value of 0.565 (95% CI 0.305-0.825) indicating a perfect test, the sensitivity and specificity of diagnosis from the ROC curve were calculated to be 70% and 80%, respectively (Figure 5).

Based on the Gene ontology (GO) function classification, the functional annotation for the genes involved in cell cycle, invasion and metastasis, angiogenesis, and signal transduction are significantly upregulated in the poor prognosis signature (annotation of genes listed in table S1). These genes include several groups for which the function annotation provides insight into the underlying biological mechanism leading to key functions involved in tumorigenesis and progression. The genes involved in the cell cycle, invasion and metastasis, angiogenesis, and signal transduction were significantly differentially expressed between two signatures (for example, GKN1, INHBA, SPP1 and THBS4). Meanwhile, unsupervised cluster analysis distinguishes between different prognostic tumors. By evaluating all 74 prognostic reporter genes, more genes belonging to these functional categories become apparent (for example, GKN1, GKN2, GIF, PSCA and LIPF).

The patients in the two groups classified by the 74 genes and the whole probes are nearly the same, except for the sample LCM, which was classified into the good prognosis group in the whole probes microarray and into the poor prognosis group in the 74-gene classification. The LCM sample was collected from the malignant tissue of a patient suffering from mucinous adenocarcinoma in stage IV with a shorter lifetime than the other patients in the formal groups. Therefore, the 74-gene classification microarray might be more reliable.

### Verification of the 74 genes custom array and correlation of the microarray data with the prognostic profile

**Figure 6:**
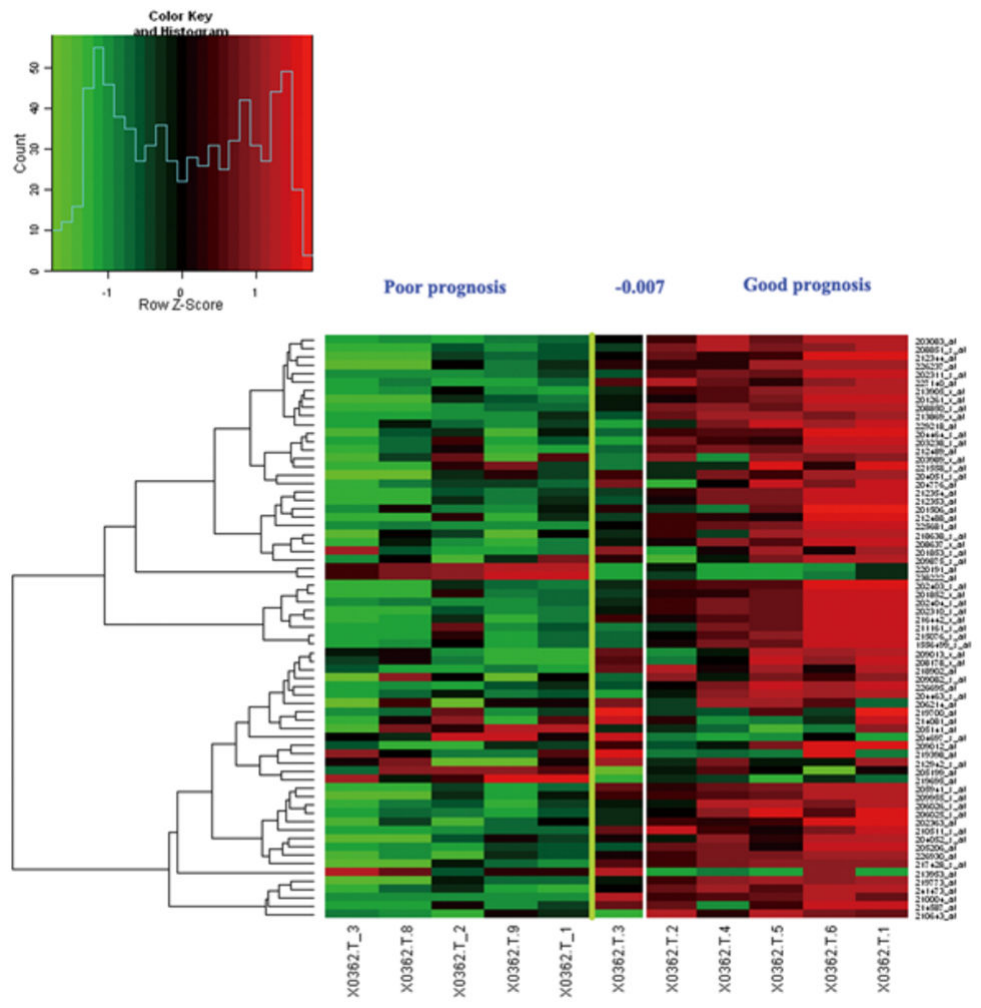
Expression data of final 74 custom microarray genes of the patients in previous study in the former data. The yellow line marks the subdivision into two dominant clusters by two-dimensional cluster analysis. The white line marks the subdivision into two dominant clusters with optimized sensitivity.

**Figure 7:**
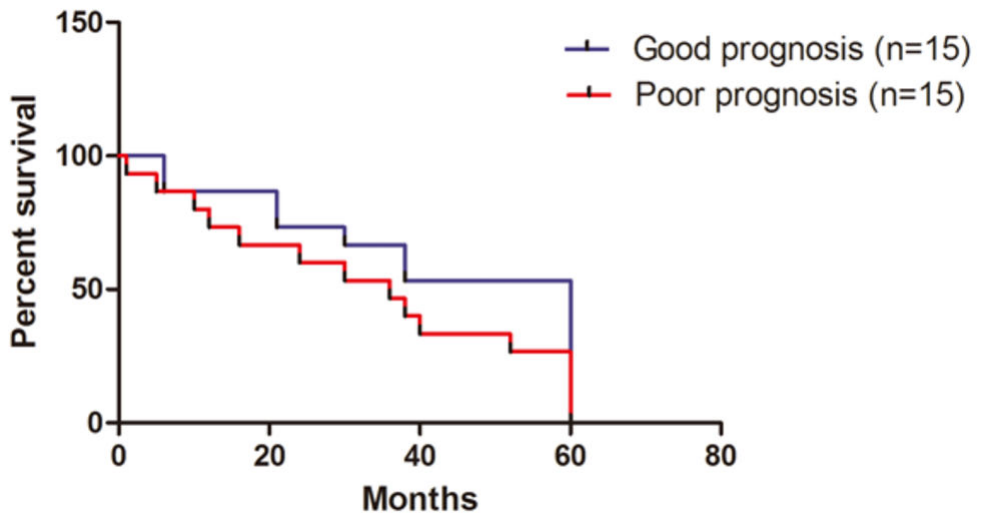
Survival curves of patients in two groups classified by the 74 genes microarray. The x-axis indicated the months within which the patients still alive. The y-axis indicated that the percentage of alive patients (including the ones with metastasis or recurrence).

For the 11 GC patients included in the previous study [12], we calculated the correlation coefficient of the level of the expression of the 74 genes with the determined average profile of these genes in tumors from patients with good prognosis (CI). A patient with a correlation coefficient of more than -0.007 (the threshold resulted in a 13 percent rate of false negative results) was then assigned to the group with the good-prognosis signature, and all other patients were assigned to the group with the poor-prognosis signature (Figure 6). In addition, the survival curve of the two groups varies markedly (p<0.05) (Figure 7). Thus, the classifier showed a comparable performance on the validation of 11 independent sporadic tumors and confirmed the predictive power and robustness of the prognosis classification of the 74-gene custom array.

### Reproducibility of customized mini-array

**Figure 8:**
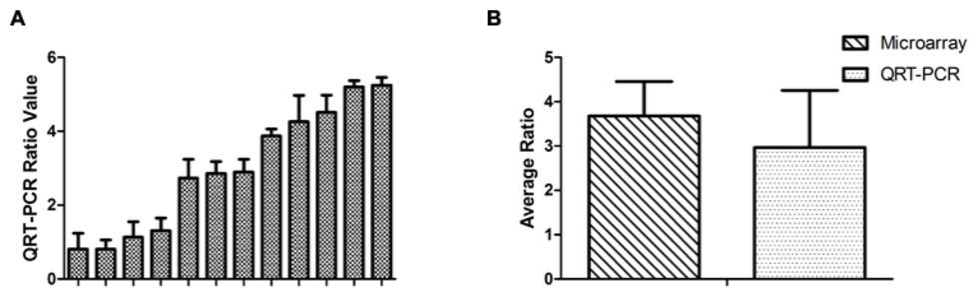
Comparison of the expression changes detected by oligonucleotide microarray and quantitative QRT-PCR analyses. The vertical numbers with log_2_ transformation are the pair-matched ratio of malignant lesions to premalignant lesions. A. The columns stand for the ratios derived from quantitative QRT- PCR experiment. B. Comparison of the ratios between microarray and quantitative QRT-PCR analyses.

To validate the data of gene expressions from the microarray data, we chose a differentiated-expression gene, INHBA, and examined its expression with quantitative RT-PCR analysis. Our data showed that the gene significantly changed expression in malignant tissues compared to premalignant tissues in 11 pair-matched samples, consistent with the results obtained from the microarray analysis (Figure 8).

### A sub group of reproduction associated genes with gastric cancer

**Figure 9:**
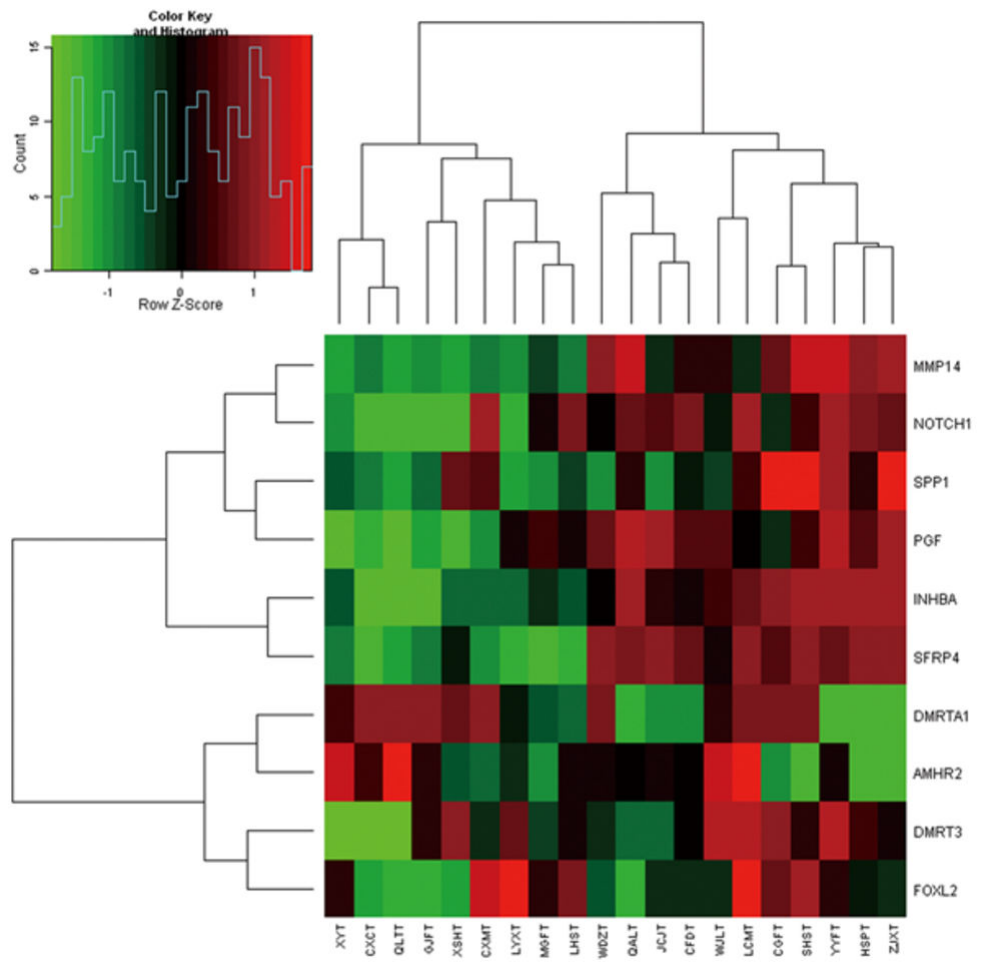
Expression data matrix of reproduction associated genes from tumors of 20 gastric cancer patients hybridized using the custom microarray. The expressions of genes between two groups have significant differences, fold change log_2_ >=1 or <= -1, P<0.01.

In the 74-gene custom microarray list, we found a group of genes with the GO classification of reproduction (Table 2), in which 5 genes for sex hormone receptors and pathways (ESRRG, DMRT3, DMRTA1, AMHR2 and FOXL2), could not only effectively separate malignant from premalignant samples but also classify poor and good prognosis with hierarchical clustering and SVM (Figure 9). In addition, two sex hormone genes had significant differentiated- expression of good prognosis to poor prognosis of GC (ESRRG 8.83, AR 0.37, p<0.01).

**Table 2:** Genes with GO classification of reproduction in 74 genes microarray group.

Symbol	Fold change	Symbol	Fold change
AMHR2	0.442	DMRT3	4.8637
INHBA	11.2072	DMRTA1	0.366
MMP14	2.009	SFRP4	13.0683
NOTCH1	2.2898	FOXL2	3.291
PGF	2.9307	SPP1	9.4141

## Discussion

In the present study, we report for the first time that the custom microarray could be an effective method for diagnosis and prediction of prognosis in GC clinically. A lack of clinical biomarkers for early gastric cancer without any specific early symptoms leads to delayed diagnosis and contributes to the high mortality of gastric cancer [23]. In some cases, changes occur only at the gene level, with no pathological change. Changes in gene expression could aid in early diagnosis, prognosis, and treatment guidance for postoperative radiation and chemotherapy. The development of microarray technologies enables the study of the possibility of pathological reversal and the evaluation and guidance of therapy. In addition, creating targeted microarray equipment might make the technique more useful. Because of the poor specificity and deficiency of mature joint diagnosis, the custom microarray has not been applied in clinical use for gastric cancer yet, although there are studies in gene diagnosis. 

Microarray analysis is a widely used technology for studying gene expression on a global scale. Several molecular assays currently employed in the clinical assessment of cancers are derived from microarray-based gene expression profiling. One example of a microarray-based assay is MammaPrint, a custom microarray of 70 genes associated with the risk of the early development of distant metastasis in young patients with lymph-node negative breast cancer. MammaPrint has been ratified by the FDA. The ability to use this profile in a high throughput diagnostic setting could be a great advantage in the prognosis and treatment of breast cancer [17-19]. However, the technology is presently not used as a routine diagnostic tool in gastric cancer, and there has been no study of custom microarrays used in the diagnosis or prediction of prognosis. In this report, we demonstrate for the first time that the custom mini-array can be used as a reliable diagnostic tool in gastric cancer. 

In this paper, we describe the development of a customized diagnostic gastric cancer mini-array and describe its reliable use in a diagnostic setting. Many clinical studies have correlated alterations in the expression of individual genes with gastric cancer outcome, often with contradictory results. Examples include CXCL1, HOXA10, and methylation of PCDH10 [24-26]. However, these genes were not included in our mini-array. It is possible that the other studies paid more attention to the functions of these genes, whereas we focused on the expressions of mRNAs. The 74-gene custom array may be a possible predictive tool for gastric cancer. The data clearly demonstrate the reproducibility and robustness of the small custom-made microarray. The use of a custom microarray could provide several advantages, such as accurate information on recurrence risk compared with conventional clinical criteria, and will thus improve the guidance for the requirement of adjuvant therapy. 

Meanwhile, because of a 2-fold higher incidence in males than in females with GC [1], several larger epidemiological surveys suggest that gender was a significant independent prognostic factor for overall survival in GC patients[27,28] and male predominance of gastric cancer correlates with a 10–15 year delay in the onset of intestinal type gastric cancer in women compared with men [29]. In this profile of the 74 gene custom mini-array, 5 genes were differentially expressed between malignant lesions and premalignant lesions of GC (ESRRG, DMRT3, DMRTA1, AMHR2 and FOXL2). This group of sex-associated genes with possible roles in GC was first proposed, and there are few studies on this group of genes associated with cancers. It is important to note that the latest study reported by Matson and colleagues showed a possible association and pathway of DMRT1, FOXL2 and the gender hormone [30]. DMRT1, DMRT3, and DMRTA1 are all included in a cluster of the gene family that have a zinc finger-like DNA-binding motif (DM domain) in common, which is also a key regulator of male development in flies and nematodes. Furthermore, the main genes in this pathway are all included in our data. The data may provide us with a research direction for sex-associated genes in GC and reveal a possible pathway and mechanism of GC canceration. 

Therefore, the array might be an excellent tool for classifying and predicting the outcome of disease in gastric cancer patients. However, there are some limitations in our profile. The samples should be expanded to verify the clinical validity and reproducibility.

## Materials and Methods

### Patients and samples

Forty surgically resected gastric cancer specimens and adjacent non-tumor specimens were obtained from Sir Run Run Shaw Hospital, School of Medicine Zhejiang University and were used during June 2007 to May 2011. We collected malignant and premalignant tissues from different regions of the resected stomach from each patient who underwent surgery. Forty grouped tissue samples from twenty patients with primary gastric cancer who underwent surgery (twenty malignant lesions, twenty premalignant lesions) were chosen for oligonucleotide microarray analysis (Table 1, detailed data in table S2). All of the collected samples were fixed, embedded, stained with H&E, and diagnosed with Lauren's and WHO classification independently by three professional pathologists. Twelve paired samples with malignant and premalignant lesions from patients who underwent surgery were chosen for quantitative reverse transcription-PCR (quantitative RT-PCR). The results from quantitative RT-PCR were compared with the pathological records from the contributing institution. Final pathological analysis was determined by consensus and reviewed if necessary. “Malignant” refers to various types of gastric cancers. “Premalignant” indicates atrophic gastritis, intestinal metaplasia and/or dysplasia. The specimens were immediately frozen in liquid nitrogen and stored at −80°C until further processing. Written informed consent was obtained before sample collection, and the study protocol was approved by the Clinical Research Ethics Committee of Sir Run Run Shaw Hospital.

### Customized Mini-Array

The original customized 8*15k mini-array contained 1042 canceration and prognosis related genes identical to the probes on the original array [12]. The mini-array included 696 differentially expressed genes between malignant lesions and premalignant lesions of GC patients that we found in previous 38, 500 gene chips, 44 collagen related genes, 54 genes for sex hormone receptors and pathways, and differentiated-expression genes found in other studies that were spotted in triplicates. Each array also includes 1042 probes for hybridization and printing quality control as well as 915 normalization genes (detailed data in table S1). Eight identical mini-arrays are present on a single 1" × 3" slide, allowing for eight individual hybridizations to be performed simultaneously (customized in Shanghai BioChip Co. Ltd., Agilent Technologies). 

### Oligonucleotide microarray

The total RNA was extracted and purified using the TRIzol Reagent (Invitrogen, USA) and RNeasy Mini Kit (Qiagen, Germany) via standard procedures recommended by the manufacturers. The levels and qualities of cRNA were measured by an Agilent 2100 Bioanalyzer (Agilent, USA), and the quality of RNA was controlled by the standard 2100 RIN>=7.0 and 28S/18S>=0.7. The cRNA was fragmented with the Gene Chip Sample Cleanup Module (Affymetrix, USA) and labeled with a single color using the Agilent enlarge notation method. Hybridization, staining, washing and scanning procedures were carried out as described in the Gene Chip Expression Analysis technical manual (Affymetrix, USA).

### Analysis of oligonucleotide microarray data

The results of the microarray were scanned by an Agilent scanner. Data were normalized and conversed after image acquisition and quantification to identify the genes with significant differential expressions using Feature Extraction software. An open source interpreted computer language (R) was used for statistical computation and graphics [12]. The raw data of custom microarray has been uploaded to the ArrayExpress as accession number A-MEXP-2338.

### Study design

We used a method based on the gene expression profiles to classify gastric cancers into prognostic or diagnostic categories. The method included the following steps: (1) design of a custom mini-array with a group of genes possibly related to GC canceration and progress based on previous studies, (2) selection of differentiated-expression genes between malignant and premalignant lesions (fold change >2 times and p< 0.05) (3), unsupervised two-dimensional cluster analysis of gene clustering and GC clustering performed independently using an agglomerative hierarchical clustering algorithm (4), selection of discriminating candidate genes from genes selected in step 2 according to their correlation with the category (good or poor prognosis) (5), determination of the optimal set of reporter genes using a leave-one-out cross validation procedure (6), prognostic or diagnostic prediction based on the gene expression of the optimal set of reporter genes [18,19], (7) GO annotation of reporter genes, and (8) analysis of correlation of the microarray data with the prognostic profile

### Quantitative RT-PCR analysis

The total RNA extracted from samples was reversely transcribed into cDNA using the prime script TM RT reagent kit (Takara, Japan) at 37°C for 15 min and at 85°C for 15 sec. PCR reactions using SYBR® premis EX Taq TM kit were performed at 95°C for 30 sec followed by 40 cycles at 95°C for 15 sec, 60°C for 10 sec, and 72°C for 40 sec with the 7500 Real-time PCR System (Applied Biosystems, USA). The housekeeping gene β-actin served as an internal control. The forward primer sequence of INHBA is GTGATGGCAAGGTCAACATCT, and the reverse one is GCGGTAGTGGTTGATGACTGT.

### Statistical analysis

We used proportional-hazards regression analysis to adjust the association between the correlation coefficient (CI) and metastases for other variables. The p-values associated with the odds ratios are calculated using Fisher's exact test.

## Supporting Information

Table S1 **The information of genes in steps of methods for designing the mini-array and the final 74 mini-array genes.**
(XLS)

Table S2 **The details of the information of lesions for microarray.**
(DOC)
