# Supplementary material for: Converting a Microarray Signature into a Diagnostic Test: A Trial of Custom 74 Gene Array for Clarification and Prediction the Prognosis of Gastric Cancer
Source: PLoS One. 2013 Dec 3;8(12):e81561. doi: 10.1371/journal.pone.0081561 (PMC3849172; doi:10.1371/journal.pone.0081561)
Supplement: Table S2 — The details of the information of lesions for microarray. (DOC) [file pone.0081561.s002.doc]

| Patients | Gender | Age | TNM | | | Stage | Pathological findings | | Lifetime (months) |
| --- | --- | --- | --- | --- | --- | --- | --- | --- | --- |
|  |  |  | T | N | S |  | T | S |  |
| CXC | M | 59 | T1 | N1 | M0 | IB | Well-differentiated adenocarcinoma | Focal mild-to-moderate atrophy (intestinal change type) | 69+ |
| CFD | M | 66 | T3 | N1 | M0 | IIIA | Poorly to moderately differentiated adenocarcinoma | Mild atrophic gastritis (intestinal change type) | 69+ |
| CGF | M | 49 | T3 | N3 | M0 | IV | Moderately differentiated adenocarcinoma | Mild atrophic gastritis (intestinal change type) | 69+ |
| CXM | M | 61 | T3 | N1 | M0 | IIIA | Well-differentiated adenocarcinoma | Moderate atrophic gastritis (intestinal change type) | 69+ |
| GJF | M | 72 | T3 | N1 | M0 | IIIA | Moderately to well differentiated adenocarcinoma, Part of the signet ring cell carcinoma | Severe atrophic gastritis (intestinal change type) | 61 |
| HSP | M | 69 | T2 | N0 | M0 | IB | Mucinous adenocarcinoma | Severe atrophic gastritis (intestinal change type) | 1 |
| JCJ | F | 59 | T3 | N3 | M0 | IV | Poorly differentiated adenocarcinoma, Part of the signet ring cell carcinoma | Mild atrophic gastritis (intestinal change type) | 52 |
| LHS | M | 67 | T3 | N2 | M0 | IIIB | Moderately differentiated adenocarcinoma | Moderate atrophic gastritis (intestinal change type) | 37 |
| LCM | M | 51 | T4 | N1 | M0 | IV | Mucinous adenocarcinoma | Moderate atrophic gastritis (intestinal change type) | 37 |
| LYX | F | 69 | T3 | N1 | M0 | IIIA | Poorly differentiated adenocarcinoma | Severe atrophic gastritis (intestinal change type) | 49 |
| MGF | M | 64 | T2 | N1 | M0 | II | Signet ring cell carcinoma, part of mucinous adenocarcinoma | Severe atrophic gastritis (intestinal change type) | 69+ |
| QAL | M | 69 | T3 | N1 | M0 | IIIA | Poorly differentiated adenocarcinoma | Moderate atrophic gastritis (intestinal change type) | 40  recurrence |
| QLT | M | 74 | T2 | N0 | M0 | IB | Well-differentiated adenocarcinoma With mucus lake formation | Mild atrophic gastritis (intestinal change type) | 69+ |
| SHS | M | 72 | T3 | N3 | M0 | IV | Moderately differentiated adenocarcinoma | Moderate atrophic gastritis (intestinal change type) | 58 |
| WDZ | M | 57 | T2 | N2 | M0 | IIIA | Poorly differentiated adenocarcinoma | Mild atrophic gastritis (intestinal change type) | 53 |
| XY | F | 49 | T2 | N0 | M0 | IB | Well-differentiated adenocarcinoma | Severe atrophic gastritis (intestinal change type) | 60 |
| XSH | F | 56 | T1 | N0 | M0 | IA | Moderately differentiated adenocarcinoma | Moderate atrophic gastritis (intestinal change type) | 52 |
| WJL | M | 57 | T3 | N2 | MX  (The left pelvic nodule) | IIIB | poorly differentiated adenocarcinoma | Moderate atrophic gastritis (intestinal change type) | 63 |
| YYF | F | 62 | T3 | N1 | M0 | IIIA | Moderately differentiated adenocarcinoma | Mild atrophic gastritis (intestinal change type) | 45 |
| ZJX | M | 59 | T3 | N2 | M0 | IIIB | Poorly differentiated adenocarcinoma, Part of the signet ring cell carcinoma | Severe atrophic gastritis (intestinal change type) | 16 |
